# Supplementary material for: Progression of Interstitial Fibrosis and Tubular Atrophy in Low Immunological Risk Renal Transplants Monitored by Sequential Surveillance Biopsies: The Influence of TAC Exposure and Metabolism
Source: J Clin Med. 2021 Jan 4;10(1):141. doi: 10.3390/jcm10010141 (PMC7796060; doi:10.3390/jcm10010141)
Supplement: Supplementary file 1 [file jcm-10-00141-s001.pdf]

Table S1. Correlation matrix of surrogate variables of tacrolimus exposure / metabolism at the time of the first biopsy. TAC-C_0 (ng/mL), tacrolimus trough levels; CV-TAC (%), coefficient of variability of tacrolimus levels; TTR (%), time in therapeutic range; above TR (%), time above therapeutic; below TR (%), time below therapeutic range; C/D (ng/mL/mg), concentration dose ratio.

	**TAC-C_0**	**CV-TAC**	**TTR**	**Above TR**	**Below TR**	**C/D**
TAC-C_0	1					
CV-TAC	**-0.226**<br>**0.037**	1				
TTR	-0.047<br>0.671	**-0.476**<br>**<0.001**	1			
Above TR	0.158<br>0.148	**0.293**<br>**0.006**	**-0.863**<br>**<0.001**	1		
Below TR	**-0.215**<br>**0.049**	**0.338**<br>**0.002**	**-0.239**<br>**0.028**	**-0.285**<br>**0.008**	1	
C/D	0.161<br>0.142	0.052<br>0.639	**-0.265**<br>**0.014**	**0.311**<br>**0.004**	-0.095<br>0.386	1

Table S2. Correlation matrix of surrogate variables of tacrolimus exposure / metabolism at the time of the second biopsy. TAC-C_0 (ng/mL), tacrolimus trough levels; CV-TAC (%), coefficient of variability of tacrolimus levels; TTR (%), time in therapeutic range; above TR (%), time above therapeutic; below TR (%), time below therapeutic range; C/D (ng/mL/mg), concentration dose ratio.

	**TAC-C_0**	**CV-TAC**	**TTR**	**Above TR**	**Below TR**	**C/D**
TAC-C_0	1					
CV-TAC	0.048<br>0.665	1				
TTR	**-0.427**<br>**<0.001**	-0.149<br>0.178	1			
Above TR	**0.230**<br>**0.005**	0.111<br>0.318	**-0.792**<br>**<0.001**	1		
Below TR	**-0.222**<br>**0.041**	**0.229**<br>**0.037**	-0.027<br>0.809	**-0.304**<br>**0.005**	1	
C/D	**0.236**<br>**0.029**	-0.006<br>0.955	-0.018<br>0.870	-0.005<br>0.962	-0.091<br>0.405	1

Table S3. Inflammation and tubulitis in areas of interstitial fibrosis / tubular atrophy in the first surveillance biopsy and surrogate variables of tacrolimus exposure / metabolism. TAC-C_0, tacrolimus trough levels; CV-TAC, coefficient of variability of tacrolimus levels; TTR, time in therapeutic range; above TR, time above therapeutic; below TR, time below therapeutic range; C/D, concentration dose ratio; i-IFTA. Interstitial inflammation in areas of interstitial fibrosis and tubular atrophy; t-IFTA, tubulitis in areas of interstitial fibrosis and tubular atrophy.

	**i-IFTA=0 & t-IFTA=0**	**i-IFTA ≥1 & t-IFTA=0**	**i-IFTA=0 & t-IFTA ≥1**	**i-IFTA=1 & t-IFTA≥1 or i-IFTA≥1 & t-IFTA=1**	**i -IFTA≥2 & t-IFTA≥2**	**p-value**
	Normal	Inflammation w/o tubulitis	Tubulitis w/o inflammation	Borderline changes	Chronic TCMR	
N	31	27	1	24	2	
TAC-C_0 (ng/mL)	10.0 ± 2.9	9.4 ± 2.1	9.2	9.4 ± 2.3	9.6 ± 1.1	0.8815
CV-TAC (%)	31 ± 12	30 ± 15	36	32 ± 12	33±14	0.9712

TTR (%)	48 ± 18	63 ± 22	60	54 ± 22	78 ± 9	0.1123
Time above TR (%)	42 ± 18	31 ± 24	24	32 ± 21	20±9	0.3659
Time below TR (%)	10 ± 14	6 ± 8	16	14 ± 16	2± 0	0.1496
C/D (ng/mL/mg)	1.8 ± 1.3	2.2 ± 1.7	1.0	2.0± 1.4	1.7 ± 0	0.7546

Table S4. Inflammation and tubulitis in areas of interstitial fibrosis / tubular atrophy in the second surveillance biopsy and surrogate variables of tacrolimus exposure / metabolism. TAC-C_0, tacrolimus trough levels; CV-TAC, coefficient of variability of tacrolimus levels; TTR, time in therapeutic range; above TR, time above therapeutic; below TR, time below therapeutic range; C/D, concentration dose ratio; i-IFTA. Interstitial inflammation in areas of interstitial fibrosis and tubular atrophy; t-IFTA, tubulitis in areas of interstitial fibrosis and tubular atrophy.

	**i-IFTA=0 & t-IFTA=0**	**i-IFTA ≥1 & t-IFTA=0**	**i-IFTA=0 & t-IFTA ≥1**	**i-IFTA=1 & t-IFTA≥1 or i-IFTA≥1 & t-IFTA=1**	**i -IFTA≥2 & t-IFTA≥2**	**p-value**
	Normal	Inflammation w/o tubulitis	Tubulitis w/o inflammation	Borderline changes	Chronic TCMR	
N	26	23	2	29	5	
TAC-C_0 (ng/mL)	8.6 ± 1.8	8.2 ± 2.0	6.7 ± 1.5	8.7 ± 2.9	8.0 ± 1.9	0.6952
CV-TAC (%)	18 ± 6	17 ± 7	22 ± 0	19 ± 8	21 ± 8	0.7380
TTR (%)	70 ± 27	63 ± 30	74 ± 37	62 ± 16	52 ± 25	0.4942
Time above TR (%)	29 ± 28	33 ± 32	26 ± 37	23 ± 27	41 ± 34	0.6598
Time below TR (%)	1 ± 3	6 ± 17	19 ± 28	4 ± 7	8 ± 13	0.1149
C/D (ng/mL/mg)	2.5 ± 1.1	2.1 ± 0.9	1.7 ± 0.8	1.9 ± 1.0	2.3 ± 0.9	0.1898
